# NAD+ and Niacin Supplementation as Possible Treatments for Glaucoma and Age-Related Macular Degeneration: A Narrative Review

**DOI:** 10.3390/nu16162795

**Published:** 2024-08-21

**Authors:** Mohamed R. Gemae, Mario D. Bassi, Patrick Wang, Eric K. Chin, David R.P. Almeida

**Affiliations:** 1 School of Medicine, Queen’s University, Kingston, ON K7L 3N6, Canada; 2 Department of Ophthalmology, Kingston Health Science Centre, Queen’s University, Kingston, ON K7L 5G2, Canada; 3 Retina Consultants of Southern California, Redlands, CA 92374, USA; 4 Erie Retina Research & Center for Advanced Surgical Exploration, Erie, PA 16507, USA

**Keywords:** nicotinamide adenine dinucleotide, anticholinergics, glaucoma, age-related macular degeneration, reactive oxygen species, retina

## Abstract

Glaucoma and age-related macular degeneration (AMD) are progressive retinal diseases characterized by increased oxidative stress, inflammation, and mitochondrial dysfunction. This review investigates the potential therapeutic benefits of NAD+ and niacin supplementation in managing glaucoma and AMD. A literature search was conducted encompassing keywords such as “niacin”, “NAD”, “glaucoma”, “AMD”, and “therapeutics”. NAD+ depletion is associated with increased oxidative stress and mitochondrial dysfunction in glaucoma and AMD. Niacin, a precursor to NAD+, has shown promise in replenishing NAD+ levels, improving choroidal blood flow, and reducing oxidative damage. Animal studies in glaucoma models indicate that nicotinamide (NAM) supplementation preserves RGC density and function. Large-scale population-based studies indicate an inverse correlation between niacin intake and glaucoma prevalence, suggesting a preventative role. Randomized controlled trials assessing niacin supplementation showed significant improvements in visual field sensitivity and inner retinal function, with a dose-dependent relationship. In AMD, nicotinamide supplementation may improve rod cell function and protect against oxidative stress-induced damage. Cross-sectional studies reveal that individuals with AMD have a lower dietary intake of niacin. Further studies suggest niacin’s role in improving choroidal blood flow and dilating retinal arterioles, potentially mitigating ischemic damage and oxidative stress in AMD. Beyond current management strategies, NAD+ and niacin supplementation may offer novel therapeutic avenues for glaucoma and AMD. Further research is warranted to elucidate their efficacy and safety in clinical settings.

## 1. Introduction

Glaucoma and age-related macular degeneration (AMD) are among the leading causes of blindness in the elderly. The most prevalent form of glaucoma, primary open-angle glaucoma (POAG), is a progressive, permanent loss of vision caused by chronic optic neuropathy involving damage to the retinal ganglion cells (RGCs) and their axons, composing the optic nerve [1]. While glaucoma typically affects most of the retinal area with associated RGCs and axons, AMD is a disease specific to the retina’s macular region, leading to a progressive loss of central-focused vision [2,3]. Regardless of their variations and progression rate, they share common etiologies, including increased oxidative stress, inflammation, mitochondrial dysfunction, and increased intraocular pressure (IOP) [4]. As both are heavily associated with increased IOP, it remains currently the only therapeutic target for glaucoma [5] and is a mainstay of treatment for both glaucoma and AMD [4]. Although IOP treatment is effective in most cases, it is possible that glaucoma can arise without the presence of elevated IOP [6].

It is clear, however, that in addition to increased IOP, there is distinct dysfunction in the oxidative phosphorylation of retinal cells in both chronic conditions. Evidence has demonstrated a depletion in nicotinamide adenine dinucleotide (NAD+) in those with glaucoma [7], which is related to normal aging [8], increasing susceptibility to POAG. Promising new research has revealed a potential role for NAD+ to improve mitochondrial dysfunction and oxidative stress as a treatment for both glaucoma and AMD [4,9]. Niacin has also demonstrated some benefits in improving choroidal blood flow in patients with AMD [10], and individuals with a higher intake of niacin in their diet have lower odds of developing glaucoma, suggesting a possible new avenue of treatment [11]. It is important to acknowledge that, despite its benefits, niacin has been associated with adverse ocular outcomes, including cystoid macular edema [12]. This underscores the complexity of niacin’s impact on ocular health and highlights the need for cautious therapeutic application. Nonetheless, this review aims to delineate the underlying pathophysiology of glaucoma and AMD and the potential protective effects that niacin and NAD+ may offer as possible therapies in their progression.

## 2. Materials and Methods

This narrative review draws upon the literature that elucidates the potential roles of NAD+ and niacin in glaucoma and AMD, which were identified through a search of the MEDLINE database. The search encompassed Medical Subject Heading (MeSH) terms and specific keywords such as “niacin”, “NAD”, “glaucoma”, “AMD”, and “therapeutics”. We included preclinical studies, case reports, case series, observational studies, interventional studies, and randomized controlled trials. References and citation lists of identified studies were manually searched (via Google Scholar).

Abstracts of the identified articles underwent binary classification for inclusion or exclusion in the review. The inclusion criteria included articles in English, peer-reviewed publications, those available in full text, and those offering an examination of NAD+ and niacin’s impact on glaucoma or AMD (or disease models). Two reviewers (MDB and MRG) screened the abstracts independently. Consensus with a third reviewer (PW) resolved articles requiring further clarification.

The full text of articles that passed the abstract screening was read, and relevant data were extracted to establish a structured framework for analysis. Specifically, we extracted the year of publication, disease process, use of niacin or derived agents, mechanism of action, and efficacy.

## 3. Overview of Oxidative Stress

### 3.1. Reactive Oxygen Species Production and Pathogenesis

Reactive oxygen species (ROS), also known as free radicals, are produced from normal metabolism as a byproduct of oxidative phosphorylation in mitochondria and from exogenous sources, such as smoking [13]. When the body’s innate ability to protect against ROS damage, called antioxidant capacity, is diminished, we see significant damage called oxidative stress. The pathophysiology of how oxidative stress harms our body is complex. ROS are reactive due to their unstable electron arrangement, where they have unpaired electrons and thus are electrophilic, scavenging any molecule available to them to become more stable [14]. Therefore, at a foundational cellular level, free ROS accumulation rapidly breaks down our body’s macromolecules, such as cell membranes, proteins, DNA, and lipid peroxidation [13,14]. If severe enough, lipid peroxidation can generate a chain reaction where, once started, all new byproducts of the ROS interaction with the lipids will also create new ROS [13]. In ocular disease caused by oxidative stress, retinal disease appears as a consequence of impaired antioxidant capacity due to the inhibited mitosis of the retinal pigment epithelium (RPE) after radiation exposure, thus increasing ROS accumulation [13].

In cases where mitochondrial efficiency declines, as seen with aging and certain pathological conditions, there is an increase in ROS production, which not only damages the mitochondria but also initiates a self-perpetuating cycle of oxidative damage that extends to other cellular components [15]. For example, other key metabolic pathways, including the tricarboxylic acid cycle and glycolysis, may also be disrupted [16]. This disruption further exacerbates oxidative stress, contributing to cellular damage.

**Figure 1:**
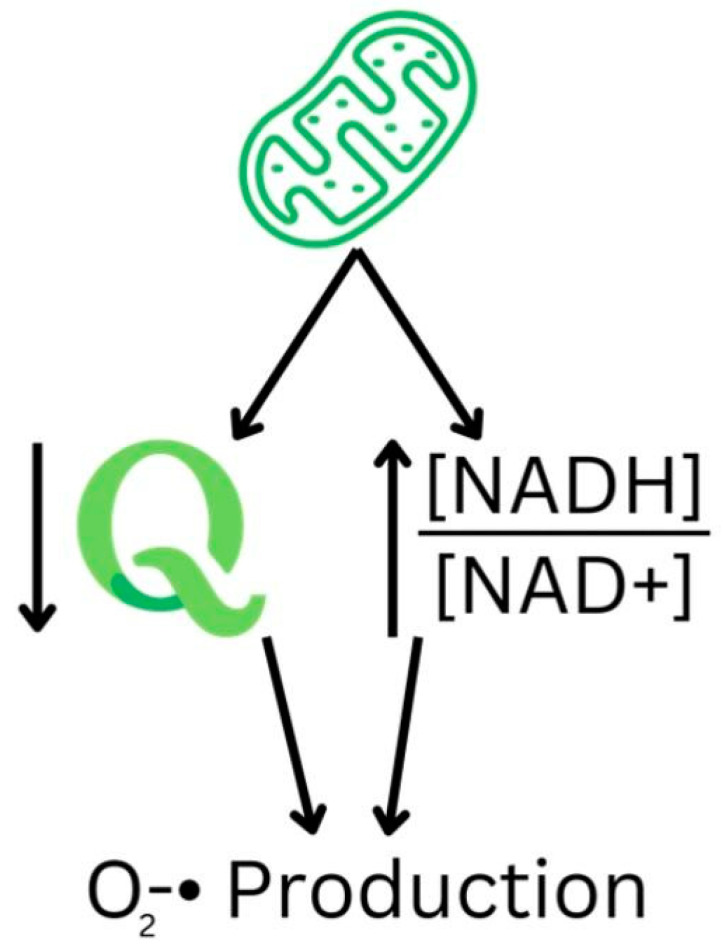
The mitochondrial states of elevated superoxide production. The reduced coenzyme Q pool and reduced NAD+ lead to increased O_2−_ production.

The most common ROS in many diseases is the superoxide anion (O_2−_), generated by various enzymes, such as NADPH oxidases, and is heavily involved in cellular oxidation. Out of the 11 sites responsible for producing ROS in the electron transport chain in the mitochondria, 6 function from the redox potential of the NADH/NAD+ isopotential pool [17]. When NADPH oxidase is inhibited, human cells experience reduced ROS production and DNA damage [18]. Specifically, mitochondria experience significant superoxide production in two states: firstly, when they are not making ATP and thus have an elevated protonmotive force with a reduced coenzyme Q (CoQ) pool, mediated by Complex I, and secondly, when there is a high NADH/NAD+ ratio in the mitochondrial matrix, thereby reducing the substrates required for efficient ATP production (Figure 1). Hence, NAD+ plays a critical role in limiting ROS production.

### 3.2. Role of NAD+ in NADPH Oxidase and ROS Production

As mentioned above, mitochondria produce superoxide excessively during periods of low NAD+ concentrations. With an aging population and those with glaucoma having reduced NAD+ serum concentrations [7,8], this increases the risk of retinal diseases due to dysfunctional oxidative phosphorylation and is a potential target for novel therapies. In human cells, niacin restriction was found to stimulate NADPH oxidase activity and increase the production of harmful ROS, damaging cell DNA, halting mitosis, limiting their growth rate, and increasing apoptosis [19]. These changes were shown to be reversed in the presence of nicotinamide or NADPH oxidase inhibitors [19]. Similarly, RGCs were found to be protected from oxidative stress during ischemia reperfusion injury following NADPH oxidase genetic deletion [20].

Furthermore, the salvage pathway of NAD+ synthesis is crucial for maintaining NAD+ levels under stress conditions [21]. NAMPT converts nicotinamide into nicotinamide mononucleotide (NMN), which is then converted into NAD+ by the enzyme NMN adenylyltransferase (NMNAT). This underscores the importance of the salvage pathway, particularly the role of NAMPT, in mitigating NAD+ depletion and preventing further mitochondrial dysfunction and ROS production [22].

Moreover, NAD+ acts as a co-substrate for sirtuins (SIRTs), a family of deacetylase enzymes that regulate mitochondrial function and oxidative stress response. For instance, SIRT3, located in mitochondria, is particularly important in maintaining mitochondrial integrity and reducing ROS production [23]. Conversely, NAD+ depletion has been linked to the reactivation of PARP enzymes, which consume NAD+ during DNA repair processes, further exacerbating NAD+ deficiency and leading to increased oxidative stress and cellular damage [24].

### 3.3. Oxidative Stress and Retinal Diseases

Dysfunctional mitochondrial oxidative phosphorylation with ROS production is associated with various organ system damage [25], including playing an extensive role in neurodegenerative diseases [26]. Chronic oxidative stress has been implicated in the accelerated aging of RPE cells, contributing to mitochondrial dysfunction, increased ROS production, and cellular senescence, all of which contribute to the pathogenesis of retinal diseases [27]. Additionally, the interplay between ROS and chronic inflammation is a critical factor in the pathogenesis of retinal diseases, with oxidative stress exacerbating inflammatory processes through the activation of transcription factors like NF-kB [28]. The progressive nature of oxidative damage in the retina underscores the importance of early intervention strategies that can mitigate ROS production and preserve retinal function [29].

Furthermore, ischemic injury to RGCs via elevated intraocular pressure was shown to elevate isotypes of NADPH oxidase, increasing ROS production and suggesting a role in RGC death [30]. Injury due to oxidative stress plays a significant role in glaucoma and the death of RGCs [31]. Oxidative stress, by overwhelming the RGCs’ antioxidative defense systems, leads to mitochondrial dysfunction and triggers the intrinsic apoptotic pathway, culminating in RGC death [32]. In glaucoma, ROS not only directly damage cellular components, but also exacerbate inflammatory responses and accelerate the degeneration of RGCs [33]. This is especially critical in the context of mitochondrial health, as RGCs are particularly vulnerable to oxidative damage due to their high metabolic demand [34]. Additionally, studies have shown that RGC axons are susceptible to oxidative stress-induced degeneration even before the appearance of clinical symptoms, indicating that oxidative stress plays an early and pivotal role in the role of pathogenesis of glaucoma [35].

In AMD specifically, we see increased oxidative stress concentrations due to ROS production proliferation and impaired antioxidant capacity [36]. Mitochondrial dysfunction in RPE cells leads to increased ROS production, which exacerbates the formation of drusen and activates the complement system, further driving chronic inflammation and cell death [37]. The impaired mitochondrial function in AMD is also associated with defective mitophagy, leading to the accumulation of damaged mitochondria and sustained oxidative damage, which drives photoreceptor degeneration [38]. Moreover, ROS-induced damage to RPE cells disrupts the blood–retina barrier, facilitating the ingress of inflammatory cells and further promoting neovascularization in wet AMD [39].

As mitochondrial Complex I is critical for ROS production, defects in the encoding genes for this complex were shown to increase endogenous ROS concentrations in the retina, lower ATP concentrations, cytochrome c release, and eventual greater apoptosis in patients with POAG [40], demonstrating a role of oxidative stress in retinal disease. The odds of glaucoma confirmed by fundus imaging are significantly lower with elevated niacin intake in adults over 40 years old [11]. Interestingly, the same study found that antioxidant administration protected against apoptosis and cytochrome c release, establishing that the damage resulted from the oxidative stress produced. Likewise, an elevated C-reactive protein (CRP) level, a common marker of systemic inflammation, has been shown to inhibit nitric oxide-mediated dilation in retinal arterioles through the stimulation of NADPH oxidases, creating superoxides, further supporting NADPH oxidases’ role in retinal diseases [41]. Finally, a murine study found that NADPH oxidases are primarily responsible for retinal vascular inflammation during acute and chronic conditions associated with retinal vascular inflammation, such as glaucoma and AMD [42].

Nicotinamide, an amide form of niacin, offers a distinct advantage in therapeutic applications. Like niacin, nicotinamide has also been shown to be directly involved in replenishing retinal NAD+ levels, thereby offering protection against oxidative stress-induced retinal damage [9]. Some studies involving NAD+ precursors such as nicotinamide have not only restored NAD+ levels, but also conferred significant protection against retinal degeneration [43]. However, due to its different molecular structure, nicotinamide does not cause some common side effects associated with niacin’s vasodilatory effect, such as flushing and itching, making it a more favorable option for long-term treatment [7].

Overall, it is clear that NADPH oxidases and NAD+ play a critical role in the pathogenesis of retinal cell apoptosis and disease states like POAG and AMD through ROS-mediated damage. Niacin and its derivatives may offer a promising therapeutic intervention avenue for patients with these conditions.

## 4. Effect of Niacin on Glaucoma

Neuroprotective therapies, including vitamins such as niacin, have garnered attention in recent years as therapeutic avenues for neurodegenerative diseases like glaucoma [44]. The potential impact of niacin on glaucoma has garnered attention in recent research due to its reported neuroprotective benefits [4,45,46]. Characterized by progressive optic nerve damage and RGC death, emerging evidence suggests that niacin supplementation could be therapeutic in addressing these structural damages in POAG [47].

Animal studies have provided compelling insights into the effects of niacin in glaucomatous eyes. In glaucoma-prone mice, the oral administration of nicotinamide (NAM) exhibited the potential to maintain RGC density despite IOP-induced stress. In addition to corroborating these findings, it was also reported that NAM supplementation can cause a transient increase in mitochondrial size and motility, assisting oxidative phosphorylation efficiency and thus reducing ROS accumulation [48]. In mice models with glaucoma, NAM supplementation has replenished NAD levels and protected against RGC loss, optic nerve damage (measured by cup area and general morphology), and mitochondrial dysfunction [49,50]. These effects may even have a dose-dependent relationship, with more excellent protection from higher doses of NAM supplementation [51]. On the other hand, nicotinamide riboside (NR) has also shown the potential to replenish NAD concentrations and preserve RGC function when injected in mice models with RGC damage [52].

Human studies have also shed light on the clinical relevance of niacin supplementation in glaucoma patients. Large-scale population-based studies have indicated an inverse correlation between niacin intake and the prevalence of glaucoma, emphasizing the preventative role of niacin [53].

Furthermore, two randomized controlled trials have been performed to assess the therapeutic efficacy of niacin supplementation. In one trial, a combination of a high dose of 3000 mg of NAM and pyruvate supplementation was given to patients for six weeks and compared with a placebo group [54]. Their results demonstrated significant improvements in visual field sensitivity in the treatment group, especially for patients with intermediate sensitivity at baseline [9]. These results suggest that niacin supplementation in humans may improve the function of RGCs with suboptimal function, but have less effect on completely damaged cells.

Another phase IV randomized clinical trial enrolled patients with different types of glaucoma and administered 1500 mg to 3000 mg of NAM or placebo for 6 weeks. As measured with an electroretinogram, they observed significant improvements in inner retinal function, with a trend toward a dose-dependent relationship [9]. IOP, visual acuity, mean arterial pressure, and retinal nerve fiber layer (RNFL) thickness showed no difference after NAM supplementation. Interestingly, there appears to be no correlation between low dietary niacin intake and age-related RNFL thinning in healthy eyes. Based on these results, Charng et al. (2022) suggest that the therapeutic efficacy of niacin on the retina and glaucoma is likely optimized at dosages surpassing the recommended dietary intake levels [47].

Both studies reported safety outcomes of NAM supplementation. An adverse effect common between studies is mild gastrointestinal discomfort, occurring in up to 31% of patients (e.g., constipation, soft stools, nausea). These side effects either self-resolved or ceased with the discontinuation of the treatment [9,54]. There was also an incidence of tinnitus in one patient who was lost to follow-up. Therefore, it is important to highlight that while a dose-dependent relationship exists for supplementation with greater benefit, healthcare providers should be cautious in recommending large doses of niacin and nicotinamides to avoid these side effects.

In sum, there is preliminary evidence from both animal and human studies supporting the protective effects of NAD+ and niacin on slowing glaucoma progression. The findings described above are summarized in Table 1.

## 5. Effect of Niacin on AMD

AMD is a leading cause of visual impairment arising from a complex interplay of risk factors like age, genetics, and diet [2,55,56]. One of the pillars of AMD management is dietary intervention to prevent disease progression and minimize visual field deficits.

A significant challenge with AMD lies in its asymptomatic phase, until it enters the intermediate stage, which is characterized by structural changes in the retinal pigment epithelium (RPE) without apparent visual deficits [3,57,58]. As described above, RPE cells are critically dependent on NAD+’s adequate availability and metabolism [59]. Photoreceptor cell health is also dependent on NAD+, and it has been strongly related to the impact on the pathogenesis of AMD [29,60]. A study performed in aged mice showed nicotinamide supplementation of up to 300 mg/kg/day prevented deficits in rod cell function [61]. These results underscore the potential of augmenting NAD+ availability through exogenous supplementation as a therapeutic avenue in AMD.

In vivo studies have unveiled 258 metabolites in the vitreous of humans with intermediate AMD as potential prognostic markers of disease progression. Interestingly, these included low nicotinamide levels in the vitreous humor of this patient population compared with matched controls [62]. A meta-analysis that assessed metabolite levels in the plasma of patients with AMD echoed these findings, delineating significant differences in plasma nicotinamide levels in patients with AMD compared with controls. These results support niacin’s potential therapeutic role in AMD management.

Subsequent studies have scrutinized the relationship between dietary niacin intake and AMD risk. These studies used 24-hour recall to assess nutrient intake; some used the Food Frequency Questionnaire as an additional objective measure. Agrón et al. (2021) found no association between dietary niacin intake and a decreased risk of AMD progression nor the appearance of large drusen, as confirmed by fundus photographs [63]. Another large-scale cross-sectional study suggested that a high dietary intake of vitamins B5 and B6 might lower the risk of advanced AMD [64]. While their results did not support the association between dietary intake of vitamin B3 and AMD progression, a recent case–control study conducted in the Czech Republic challenged these assertions. According to their findings, individuals with AMD exhibited notably reduced dietary energy compared to their control counterparts, including lower levels of niacin consumption [65]. Additionally, their research suggests that AMD patients were less likely to meet the recommended dietary intakes of micronutrients.

Reports of niacin supplementation in humans with AMD are limited, with a lack of recent clinical evidence being published. Metelitsina and colleagues (2004) investigated the effects of choroidal blood flow in AMD, revealing transient increases of up to 24% in choroidal blood volume without affecting blood flow [10]. The same group of researchers also published results highlighting niacin’s potential in dilating retinal arterioles [66]. Therefore, niacin may assist in preventing or limiting the progression of AMD not only through diminishing ROS-induced oxidative stress on RGCs, but also through improvements in perfusion and reducing ischemic damage. Regardless, current conclusive evidence regarding the preventive or therapeutic efficacy of vitamin supplements, including vitamin B3 supplementation, in preventing or slowing down early signs of AMD remains elusive [67].

Overall, while the evidence suggests that niacin supplementation may have potential benefits in AMD management, as summarized in Table 2, the current clinical data remain inconclusive.

## 6. Conclusions

In conclusion, nicotinamide and NAD+ supplementation may play a beneficial preventative role in glaucoma and AMD development while providing novel avenues for glaucoma and AMD treatment that do not pertain solely to IOP management. Niacin and its derivatives have been tested through in vitro, in vivo, and preclinical studies, but only a few studies have tested their effectiveness in clinical studies.

Continued research efforts are warranted to elucidate the nuanced role of niacin and other dietary interventions in managing glaucoma and AMD.

## Figures and Tables

**Table 1:** A summary of the potential effects of NAD+ and niacin on glaucoma.

Effect	Study Type	Details/Findings
Protection against retinal ganglion cell (RGC) loss	Animal studies	Nicotinamide (NAM) supplementation maintains RGC density and function in glaucoma-prone mice [49,50,51,52].
Preservation of mitochondrial function	Animal studies	NAM supplementation transiently increases mitochondrial size and motility, assisting oxidative phosphorylation [49,50].
Reduction in ROS production	In vitro and animal studies	NADPH oxidase inhibition or NAD+ replenishment reduces ROS production and oxidative stress [9,55].
Prevention of RGC apoptosis	In vitro studies	NADPH oxidase inhibition protects RGCs from oxidative stress and apoptosis [17,18,19,20,25].
Inverse correlation with glaucoma prevalence	Population-based studies	A high dietary niacin intake is associated with a lower risk of developing glaucoma [11,54].
Improvement in visual field sensitivity	Randomized controlled trial	High-dose NAM and pyruvate supplementation improves visual field sensitivity in patients with glaucoma [19,20].
Reported side effects of NAM supplementation	Randomized controlled trial	NAM supplementation may cause mild gastrointestinal discomfort, with a dose-dependent relationship [9,55].

**Table 2:** A summary of the potential effects of NAD+ and niacin on age-related macular degeneration (AMD).

Effect	Study Type	Details/Findings
Improvement in rod cell function	Animal studies	Nicotinamide supplementation prevented deficits in rod cell function in aged mice [62].
Protection against oxidative stress	In vitro and animal studies	NAD+ supplementation may protect retinal cells from oxidative stress and improve RPE health [29,60].
Increases in choroidal blood flow	Human studies	Niacin transiently increased choroidal blood volume in patients with AMD [10].
Lower niacin levels in AMD patients	Case–control studies	Patients with AMD had a lower dietary intake of niacin compared to controls [63,66].
Potential retinal arteriole dilation	Human studies	Niacin may assist in dilating retinal arterioles, potentially mitigating ischemic damage in AMD [64,65].
No association with AMD progression	Population-based studies	Some studies found no association between dietary niacin intake and a decreased risk of AMD progression [67].

## Data Availability

Not applicable.

## References

[1] Wentz S.M., Kim N.J., Wang J., Amireskandari A., Siesky B., Harris A. (2014). Novel therapies for open-angle glaucoma. F1000Prime Rep..

[2] Mitchell P., Liew G., Gopinath B., Wong T.Y. (2018). Age-related macular degeneration. Lancet.

[3] Lim L.S., Mitchell P., Seddon J.M., Holz F.G., Wong T.Y. (2012). Age-related macular degeneration. Lancet.

[4] Cimaglia G., Votruba M., Morgan J.E., Andre H., Williams P.A. (2020). Potential Therapeutic Benefit of NAD(+) Supplementation for Glaucoma and Age-Related Macular Degeneration. Nutrients.

[5] Almasieh M., Wilson A.M., Morquette B., Cueva Vargas J.L., Di Polo A. (2012). The molecular basis of retinal ganglion cell death in glaucoma. Prog. Retin. Eye Res..

[6] Lee D.A., Higginbotham E.J. (2005). Glaucoma and its treatment: A review. Am. J. Health Syst. Pharm..

[7] Kouassi Nzoughet J., Chao de la Barca J.M., Guehlouz K., Leruez S., Coulbault L., Allouche S., Bocca C., Muller J., Amati-Bonneau P., Gohier P. (2019). Nicotinamide Deficiency in Primary Open-Angle Glaucoma. Investig. Ophthalmol. Vis. Sci..

[8] Mattson M.P., Arumugam T.V. (2018). Hallmarks of Brain Aging: Adaptive and Pathological Modification by Metabolic States. Cell Metab..

[9] Hui F., Tang J., Williams P.A., McGuinness M.B., Hadoux X., Casson R.J., Coote M., Trounce I.A., Martin K.R., van Wijngaarden P. (2020). Improvement in inner retinal function in glaucoma with nicotinamide (vitamin B3) supplementation: A crossover randomized clinical trial. Clin. Exp. Ophthalmol..

[10] Metelitsina T.I., Grunwald J.E., DuPont J.C., Ying G.S. (2004). Effect of niacin on the choroidal circulation of patients with age related macular degeneration. Br. J. Ophthalmol..

[11] Taechameekietichai T., Chansangpetch S., Peerawaranun P., Lin S.C. (2021). Association between Daily Niacin Intake and Glaucoma: National Health and Nutrition Examination Survey. Nutrients.

[12] Dajani H.M., Lauer A.K. (2006). Optical coherence tomography findings in niacin maculopathy. Can. J. Ophthalmol..

[13] Murphy M.P. (2009). How mitochondria produce reactive oxygen species. Biochem. J..

[14] Ray P.D., Huang B.W., Tsuji Y. (2012). Reactive oxygen species (ROS) homeostasis and redox regulation in cellular signaling. Cell. Signal..

[15] Verdin E. (2015). NAD^+^ in aging, metabolism, and neurodegeneration. Science.

[16] Magni G., Amici A., Emanuelli M., Orsomando G., Raffaelli N., Ruggieri S. (2004). Enzymology of NAD+ homeostasis in man. Cell. Mol. Life Sci. (CMLS).

[17] Zhang B., Pan C., Feng C., Yan C., Yu Y., Chen Z., Guo C., Wang X. (2022). Role of mitochondrial reactive oxygen species in homeostasis regulation. Redox Rep..

[18] Emmert H., Fonfara M., Rodriguez E., Weidinger S. (2020). NADPH oxidase inhibition rescues keratinocytes from elevated oxidative stress in a 2D atopic dermatitis and psoriasis model. Exp. Dermatol..

[19] Benavente C.A., Jacobson E.L. (2008). Niacin restriction upregulates NADPH oxidase and reactive oxygen species (ROS) in human keratinocytes. Free Radic. Biol. Med..

[20] Yokota H., Narayanan S.P., Zhang W., Liu H., Rojas M., Xu Z., Lemtalsi T., Nagaoka T., Yoshida A., Brooks S.E. (2011). Neuroprotection from retinal ischemia/reperfusion injury by NOX2 NADPH oxidase deletion. Investig. Ophthalmol. Vis. Sci..

[21] Revollo J.R., Grimm A.A., Imai S.-I. (2004). The NAD Biosynthesis Pathway Mediated by Nicotinamide Phosphoribosyltransferase Regulates Sir2 Activity in Mammalian Cells. J. Biol. Chem..

[22] Jadeja R.N., Powell F.L., Jones M.A., Fuller J., Joseph E., Thounaojam M.C., Bartoli M., Martin P.M. (2018). Loss of NAMPT in aging retinal pigment epithelium reduces NAD+ availability and promotes cellular senescence. Aging.

[23] Lautrup S., Sinclair D.A., Mattson M.P., Fang E.F. (2019). NAD+ in Brain Aging and Neurodegenerative Disorders. Cell Metab..

[24] Cantó C., Keir, Auwerx J. (2015). NAD+ Metabolism and the Control of Energy Homeostasis: A Balancing Act between Mitochondria and the Nucleus. Cell Metab..

[25] Pham-Huy L.A., He H., Pham-Huy C. (2008). Free radicals, antioxidants in disease and health. Int. J. Biomed. Sci..

[26] Kausar S., Wang F., Cui H. (2018). The Role of Mitochondria in Reactive Oxygen Species Generation and Its Implications for Neurodegenerative Diseases. Cells.

[27] Kaarniranta K., Uusitalo H., Blasiak J., Felszeghy S., Kannan R., Kauppinen A., Salminen A., Sinha D., Ferrington D. (2020). Mechanisms of mitochondrial dysfunction and their impact on age-related macular degeneration. Prog. Retin. Eye Res..

[28] Jarrett S.G., Boulton M.E. (2012). Consequences of oxidative stress in age-related macular degeneration. Mol. Asp. Med..

[29] Fisher C.R., Ferrington D.A. (2018). Perspective on AMD Pathobiology: A Bioenergetic Crisis in the RPE. Investig. Opthalmol. Vis. Sci..

[30] Dvoriantchikova G., Grant J., Santos A.R., Hernandez E., Ivanov D. (2012). Neuronal NAD(P)H oxidases contribute to ROS production and mediate RGC death after ischemia. Investig. Ophthalmol. Vis. Sci..

[31] Ahmad A., Ahsan H. (2020). Biomarkers of inflammation and oxidative stress in ophthalmic disorders. J. Immunoass. Immunochem..

[32] Tezel G. (2006). Oxidative stress in glaucomatous neurodegeneration: Mechanisms and consequences. Prog. Retin. Eye Res..

[33] Qu J., Wang D., Grosskreutz C.L. (2010). Mechanisms of retinal ganglion cell injury and defense in glaucoma. Exp. Eye Res..

[34] Osborne N.N. (2010). Mitochondria: Their role in ganglion cell death and survival in primary open angle glaucoma. Exp. Eye Res..

[35] Kamel K., Farrell M., O’Brien C. (2017). Mitochondrial dysfunction in ocular disease: Focus on glaucoma. Mitochondrion.

[36] Yildirim Z., Ucgun N.I., Yildirim F. (2011). The role of oxidative stress and antioxidants in the pathogenesis of age-related macular degeneration. Clinics.

[37] Zhu Y., Zhao K.-K., Tong Y., Zhou Y.-L., Wang Y.-X., Zhao P.-Q., Wang Z.-Y. (2016). Exogenous NAD+ decreases oxidative stress and protects H2O2-treated RPE cells against necrotic death through the up-regulation of autophagy. Sci. Rep..

[38] Hyttinen J.M.T., Viiri J., Kaarniranta K., Błasiak J. (2018). Mitochondrial quality control in AMD: Does mitophagy play a pivotal role?. Cell. Mol. Life Sci..

[39] Datta S., Cano M., Ebrahimi K., Wang L., Handa J.T. (2017). The impact of oxidative stress and inflammation on RPE degeneration in non-neovascular AMD. Prog. Retin. Eye Res..

[40] He Y., Leung K.W., Zhang Y.H., Duan S., Zhong X.F., Jiang R.Z., Peng Z., Tombran-Tink J., Ge J. (2008). Mitochondrial complex I defect induces ROS release and degeneration in trabecular meshwork cells of POAG patients: Protection by antioxidants. Investig. Ophthalmol. Vis. Sci..

[41] Nagaoka T., Kuo L., Ren Y., Yoshida A., Hein T.W. (2008). C-reactive protein inhibits endothelium-dependent nitric oxide-mediated dilation of retinal arterioles via enhanced superoxide production. Investig. Ophthalmol. Vis. Sci..

[42] Al-Shabrawey M., Rojas M., Sanders T., Behzadian A., El-Remessy A., Bartoli M., Parpia A.K., Liou G., Caldwell R.B. (2008). Role of NADPH oxidase in retinal vascular inflammation. Investig. Ophthalmol. Vis. Sci..

[43] Williams P.A., Harder J.M., Foxworth N.E., Cochran K.E., Philip V.M., Porciatti V., Smithies O., John S.W.M. (2017). Vitamin B <sub>3</sub> modulates mitochondrial vulnerability and prevents glaucoma in aged mice. Science.

[44] D’Angelo A., Vitiello L., Lixi F., Abbinante G., Coppola A., Gagliardi V., Pellegrino A., Giannaccare G. (2024). Optic Nerve Neuroprotection in Glaucoma: A Narrative Review. J. Clin. Med..

[45] Tribble J.R., Otmani A., Sun S., Ellis S.A., Cimaglia G., Vohra R., Jöe M., Lardner E., Venkataraman A.P., Domínguez-Vicent A. (2021). Nicotinamide provides neuroprotection in glaucoma by protecting against mitochondrial and metabolic dysfunction. Redox Biol..

[46] Chou T.-H., Romano G.L., Amato R., Porciatti V. (2020). Nicotinamide-Rich Diet in DBA/2J Mice Preserves Retinal Ganglion Cell Metabolic Function as Assessed by PERG Adaptation to Flicker. Nutrients.

[47] Charng J., Ansari A.S., Bondonno N.P., Hunter M.L., O’Sullivan T.A., Louca P., Hammond C.J., Mackey D.A. (2022). Association between dietary niacin and retinal nerve fibre layer thickness in healthy eyes of different ages. Clin. Exp. Ophthalmol..

[48] Ramdas W., Schouten J., Webers C. (2018). The Effect of Vitamins on Glaucoma: A Systematic Review and Meta-Analysis. Nutrients.

[49] Lee S., Van Bergen N.J., Kong G.Y., Chrysostomou V., Waugh H.S., O’Neill E.C., Crowston J.G., Trounce I.A. (2011). Mitochondrial dysfunction in glaucoma and emerging bioenergetic therapies. Exp. Eye Res..

[50] Katsyuba E., Romani M., Hofer D., Auwerx J. (2020). NAD+ homeostasis in health and disease. Nat. Metab..

[51] Williams P.A., Harder J.M., Cardozo B.H., Foxworth N.E., John S.W.M. (2018). Nicotinamide treatment robustly protects from inherited mouse glaucoma. Commun. Integr. Biol..

[52] Zhang X., Zhang N., Chrenek M.A., Girardot P.E., Wang J., Sellers J.T., Geisert E.E., Brenner C., Nickerson J.M., Boatright J.H. (2021). Systemic Treatment with Nicotinamide Riboside Is Protective in Two Mouse Models of Retinal Ganglion Cell Damage. Pharmaceutics.

[53] Jung K., Kim Y., Park C. (2018). Dietary Niacin and Open-Angle Glaucoma: The Korean National Health and Nutrition Examination Survey. Nutrients.

[54] De Moraes C.G., John S.W.M., Williams P.A., Blumberg D.M., Cioffi G.A., Liebmann J.M. (2022). Nicotinamide and Pyruvate for Neuroenhancement in Open-Angle Glaucoma. JAMA Ophthalmol..

[55] Shin H.-T., Yoon B.W., Seo J.H. (2021). Comparison of risk allele frequencies of single nucleotide polymorphisms associated with age-related macular degeneration in different ethnic groups. BMC Ophthalmol..

[56] Stahl A. (2020). The Diagnosis and Treatment of Age-Related Macular Degeneration. Dtsch. Ärzteblatt Int..

[57] Wong W.L., Su X., Li X., Cheung C.M.G., Klein R., Cheng C.-Y., Wong T.Y. (2014). Global prevalence of age-related macular degeneration and disease burden projection for 2020 and 2040: A systematic review and meta-analysis. Lancet Glob. Health.

[58] Flaxel C.J., Adelman R.A., Bailey S.T., Fawzi A., Lim J.I., Vemulakonda G.A., Ying G.-S. (2020). Age-Related Macular Degeneration Preferred Practice Pattern^®^. Ophthalmology.

[59] Jadeja R.N., Thounaojam M.C., Bartoli M., Martin P.M. (2020). Implications of NAD+ Metabolism in the Aging Retina and Retinal Degeneration. Oxidative Med. Cell. Longev..

[60] Pawlowska E., Szczepanska J., Koskela A., Kaarniranta K., Blasiak J. (2019). Dietary Polyphenols in Age-Related Macular Degeneration: Protection against Oxidative Stress and Beyond. Oxidative Med. Cell. Longev..

[61] Mills K.F., Yoshida S., Stein L.R., Grozio A., Kubota S., Sasaki Y., Redpath P., Migaud M.E., Apte R.S., Uchida K. (2016). Long-Term Administration of Nicotinamide Mononucleotide Mitigates Age-Associated Physiological Decline in Mice. Cell Metab..

[62] Yoon C.-K., Kim Y.A., Park U.C., Kwon S.-H., Lee Y., Yoo H.J., Seo J.H., Yu H.G. (2023). Vitreous Fatty Amides and Acyl Carnitines Are Altered in Intermediate Age-Related Macular Degeneration. Investig. Opthalmol. Vis. Sci..

[63] Agrón E., Mares J., Clemons T.E., Swaroop A., Chew E.Y., Keenan T.D.L. (2021). Dietary Nutrient Intake and Progression to Late Age-Related Macular Degeneration in the Age-Related Eye Disease Studies 1 and 2. Ophthalmology.

[64] Merle B.M.J., Barthes S., Féart C., Cougnard-Grégoire A., Korobelnik J.-F., Rougier M.-B., Delyfer M.-N., Delcourt C. (2022). B Vitamins and Incidence of Advanced Age-Related Macular Degeneration: The Alienor Study. Nutrients.

[65] Kráľová J.Š., Kolář P., Kapounová Z., Veselý P., Derflerová Brázdová Z. (2023). Dietary habits and dietary nutrient intake in patients with age-related macular degeneration: A case-control study. Cent. Eur. J. Public Health.

[66] Barakat M.R., Metelitsina T.I., Dupont J.C., Grunwald J.E. (2006). Effect of Niacin on Retinal Vascular Diameter in Patients with Age-Related Macular Degeneration. Curr. Eye Res..

[67] Evans J.R., Lawrenson J.G. (2017). Antioxidant vitamin and mineral supplements for preventing age-related macular degeneration. Cochrane Database Syst. Rev..

